# Supplementary material for: Inclusion Formation and Toxicity of the ALS Protein RGNEF and Its Association with the Microtubule Network
Source: Int J Mol Sci. 2020 Aug 5;21(16):5597. doi: 10.3390/ijms21165597 (PMC7460592; doi:10.3390/ijms21165597)
Supplement: Supplementary file 1 [file ijms-21-05597-s001.docx]

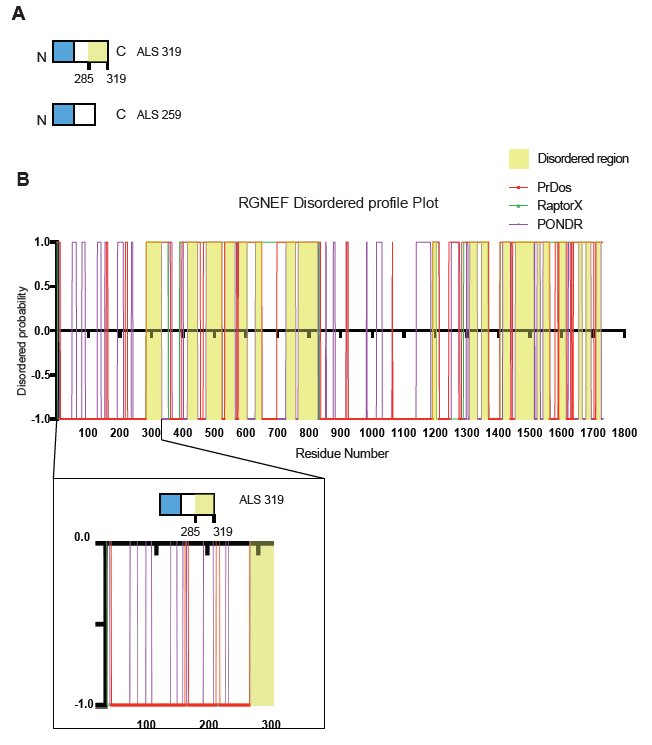


**Supplementary Figure S1. A** ALS truncated products illustration showing that the longer variant contains a domain predicted to be intrinsically disordered. **B** RGNEF disordered profile plot shows the prediction of intrinsically disordered regions within the RGNEF protein as predicted by three independent algorithms (PrDos, RaptorX, PONDR). Areas highlighted in yellow show intrinsically disordered residues predicted by all three algorithms. ALS 319 contains intrinsically disordered residues starting from 285 – 319.
